# Anticancer potential of dietary fiber for colorectal carcinoma: a narrative review

**DOI:** 10.1080/07853890.2025.2583756

**Published:** 2025-11-17

**Authors:** Muhammad Barkaat Hussain, Abdullah Faisal Albukhari, Misbahuddin Rafeeq, Muhammad Imran, Ali Hassan Albagholi, Mohammed Abdulsalam Assedoodi

**Affiliations:** a Department of Microbiology, Faculty of Medicine in Rabigh, King Abdulaziz University (KAU), Jeddah, Saudi Arabia; b Faculty of Medicine, King Abdulaziz University, Saudi Arabia; c Basic Medical Sciences, College of Medicine, Dhofar University, Oman; d Department of Surgery, Faculty of Medicine, King Abdulaziz University, Saudi Arabia

**Keywords:** Anticancer, dietary fiber, colorectal carcinoma, high-fiber diet, beta glucan, pectin, arabinoxylan

## Abstract

**Introduction:**

Dietary fiber has emerged as a preventive and adjuvant therapy for patients with colorectal carcinoma (CRC) because it possesses chemoprotective and direct anticancer properties, including the ability to modulate multiple cellular and molecular targets implicated in tumor cell survival, proliferation, invasion, angiogenesis, and immunomodulation. This review summarizes the latest epidemiological, preclinical, and clinical research on dietary fiber as a preventive and adjuvant therapy for CRC patients, focusing on the cellular and molecular mechanisms of the bioactive compounds present in or produced by fiber metabolism.

**Materials and methods:**

A structured literature search was conducted in PubMed using a Boolean search strategy to identify relevant studies on the association between dietary fiber and CRC. Studies unrelated to dietary fiber or CRC, non-peer-reviewed materials, and articles with insufficient methodological details were excluded to maintain the focus and quality of the review.

**Results:**

An overall body of epidemiological evidence suggests that the consumption of foods rich in dietary fiber or whole grains is associated with a reduced risk of CRC. Preclinical studies conducted *in vitro* and in animal models strongly support the hypothesis that dietary fiber is effective in preventing and treating CRC and provide mechanistic insights, indicating that diverse types of fiber, such as β-glucan, resistant starch, pectin, and arabinoxylan, have distinct but complementary effects. However, the findings of the clinical trials are inconclusive. This disconnect may stem from the differences in methodologies, fiber types, dosages, intervention durations, and endpoints across studies.

**Conclusions:**

According to the current evidence, dietary fiber appears to have a protective effect against the development of CRC. However, more targeted and prospectively planned RCTs using standardized dosages, formulations and combinations of dietary fiber are needed to provide additional evidence in favor of the recommendation of a high-fiber diet for the prevention and adjuvant treatment of CRC.

## 1. Introduction

Colorectal carcinoma (CRC) is the third most common cancer globally, accounting for approximately 10% of all cancer cases. It has also been identified as the second most common cause of cancer-related deaths on the global scale [1]. People living in developed countries have a greater risk of CRC emphasizing the strong connection between lifestyle factors and disease development [2]. A study revealed that, among the 11 million deaths worldwide attributed to dietary factors, approximately 1 million were linked to inadequate dietary fiber intake, which was specifically defined as less than 25 to 30 grams per day [3]. According to another study comprising seven prospective cohort studies, every 10-g daily increase in dietary fiber consumption resulted in an 11% decrease (95% CI 0.85–0.92) in all-cause death. The consumption of cereal fiber seems to have the strongest inverse correlation with mortality [4].

Although CRC ranked 4th in 1998, it is now the leading cause of cancer-related death in men and the second leading cause in women under 50, following breast cancer [5]. It is anticipated that by 2040, there will be 3.2 million new CRC cases each year (a 63% increase) and approximately 1.6 million fatalities annually (a 73% increase) [6]. The risk of CRC is linked to both non-modifiable risk factors, such as age and heredity, and modifiable lifestyle factors [7]. In the United States, approximately 50 to 60% of newly diagnosed CRC cases can be linked to lifestyle factors [8]. Several factors can increase the risk of CRC. These include a high intake of animal fats, processed meats, refined grains, and foods high in sugar content, combined with a low intake of fruits and vegetables. A lack of physical activity, smoking, being overweight and drinking too much alcohol increases this risk [9]. According to the World Cancer Research Fund nearly 50% of CRC cases are preventable by diet and lifestyle changes [10].

Patients diagnosed with CRC continue to have low 5-year survival rates despite advancement in treatment options [11]. The most effective measure against CRC remains surgery, especially in patients with localized diseases. Based on the stage and particular morphological features of the tumor, it is frequently combined with adjuvant chemotherapy, radiotherapy, immunotherapy and targeted therapy [12]. The chemotherapy for metastatic colorectal cancer (mCRC) only has 10% response rate at 5 years, signifying the limited value of standard regimen [13]. The development of drug resistance, persistent cancer stem cells and high intratumoral heterogeneity can be the reason which leads to a suboptimal response [14,15]. Drug resistance occurs through many mechanisms, including drug efflux due to increased drug excretion, mutation of drug targets to decrease drug reuptake, alteration in drug metabolism, initiation of autophagy, or ecDNA-mediated oncogene amplification [16]. The emergence of multidrug resistance in CRC in response to various anticancer treatments is another alarming aspect of chemotherapy failure [17]. In addition, standard CRC treatments can cause side effects like bone marrow depression, GI tract disturbance, neurotoxicity and nephrotoxicity [18]. As a result, these factors render mCRC difficult to manage and underscore the importance of effective treatment. In other words, it is imperative to evaluate new, better nontoxic compounds which would act either as alternative or in combination with already existing drugs. The goal is to minimize side effects, delay the emergence of drug resistance, and enhance tumor response to current treatments. Multiple treatment options have been proposed to overcome these cancer resistance mechanisms [19,20].

One potential therapeutic option is dietary intervention, particularly increased fiber intake, which has become a key strategy for minimizing the risk and progression of CRC through its impact on the gut microbiota, intestinal tissue homeostasis and intestinal immunity, as supported by numerous studies [21,22]. Importantly, the current evidence regarding the health impacts of dietary fiber comes from both epidemiological and intervention studies [23]. The results of these studies are consistent when analyzed together, ruling out the possibility of a coincidental or random effect [3]. Dietary fibers, including β-glucan, resistant starch, pectin, arabinoxylan, dextrin, and galacto-oligosaccharides, possess both chemopreventive and direct anticancer properties [24–26]. They achieve this goal by modulating various molecular targets and multiple signaling pathways related to cancer cell survival, proliferation, invasion, angiogenesis, and immune modulation [22,27]. Notably, some natural compounds derived from dietary fiber have demonstrated additive or synergistic effects, as well as reduced side effects, when used alongside standard cytotoxic treatment in patients with CRC, suggesting their potential role as preventive and adjuvant therapies [28,29].

The literature reveals a significant gap in the clinical translation of the preventive and adjuvant benefits of dietary fiber for CRC. Although extensive epidemiological and preclinical evidence underscores the chemoprotective and anticancer properties of dietary fiber, clinical trials often yield inconclusive or inconsistent results. This disconnect may stem from various methodologies, such as differences in fiber types, dosages, intervention durations, and endpoint assessments used across studies. Furthermore, challenges such as participant adherence, insufficient follow-up periods and self-reported dietary assessments compromise data accuracy. Addressing these gaps is crucial because dietary fiber represents a low-cost non-toxic intervention with the potential to enhance CRC management. This review is essential for consolidating existing evidence, identifying methodological limitations, and proposing strategies to optimize clinical trials, ultimately guiding the integration of dietary fiber into preventive and therapeutic regimens for CRC.

## 2. Methodology

A structured literature search was conducted in PubMed using a Boolean search strategy to identify relevant studies on the association between dietary fiber and CRC. Two independent researchers (MBH, MI) conducted the search. The search string used was: (‘Dietary Fiber”[Mesh]) AND “Colorectal Neoplasms”[Mesh], which restricted the search to article titles to ensure a focused selection of studies directly addressing the topic. This approach allowed for the retrieval of peer-reviewed articles discussing the role of dietary fiber in the prevention, progression, and adjuvant treatment of CRC. Additional databases, such as Google Scholar, were searched to identify relevant articles that may not have been captured in the initial search from PubMed. The search results were screened on the basis of the inclusion criteria, and the exclusion criteria and relevant studies were critically appraised to synthesize the key findings.

The selection criteria for this study emphasized epidemiological studies, preclinical studies, and clinical trials in which dietary fiber was employed as a preventive strategy or as part of the treatment for CRC. A rigorous evaluation of the identified articles was performed by examining the title, abstract, and full text to ensure the integrity of data extraction. A preliminary review of titles and abstracts was subsequently conducted to eliminate irrelevant articles that may have been included in the initial search. The complete content of the remaining articles was then reviewed, and their reference lists were scrutinized to identify relevant current studies not captured during the initial search process. The inclusion and exclusion criteria were applied to refine the resulting set and ensure that only studies relevant to the review were considered. The literature was critically appraised to identify key themes, trends, and gaps. The synthesis was conducted descriptively, highlighting the conceptual and empirical advancements in the field. This review does not include a systematic appraisal of the literature or meta-analysis but aims to provide a cohesive narrative on the current state of evidence in this field.

### 2.1. Inclusion and exclusion criteria

For inclusion in the review, there were no restrictions on the year of publication; however, the selected studies had to be written in English and had to address the effects of dietary fiber on the prevention, progression, or treatment of CRC as adjuvant therapy. Studies published in languages other than English were excluded. Additionally, case reports, trial protocols, editorial opinions, conference proceedings, and conference abstracts were eliminated, as these types of publications were deemed to lack comprehensive information about the studies. Any study that failed to meet at least one inclusion criterion was excluded. Nonpeer-reviewed materials and articles lacking sufficient methodological details were also excluded to ensure that the review maintained a high standard of focus and quality.

## 3. Epidemiological evidence

Over the past several decades, several epidemiological studies have established important connections between dietary habits and the risk of developing CRC [30]. The consumption of a fiber-rich diet, calcium, milk, or whole grains is associated with a significantly decreased risk of CRC, whereas a diet rich in red meat and processed meats significantly elevates the risk of developing this disease [31]. Notably, there is considerable evidence supporting the protective effects of dietary fiber and whole grains, suggesting that these components may play a vital role in CRC [32]. The American Institute for Cancer Research and the World Cancer Research Fund collaborated to produce a summary report that analyzed the relationship between dietary exposures and CRC *via* a dose-response meta-analysis and systematic reviews. Substantial evidence indicates that the intake of foods rich in dietary fiber or whole grains is associated with a decreased risk of CRC [33]. In addition, a systematic review and meta-analysis comprising 99 studies, investigated the relationships among dietary fiber, whole grains, and CRC risk. The review concluded that CRC risk was significantly reduced by dietary fiber intake. If an individual consumes an extra 10 grams of dietary fiber (per day) on a daily basis, it reduces the chances of having CRC by 10%. CRC risk was most strongly associated with total dietary fiber derived from cereals. Adding more whole grains to the diet can cut the risk significantly by 22% for every additional 90 g eaten each day [34].

An umbrella review examined 45 meta-analyses originating from prospective studies together providing more than 100 adjusted summary risk estimates. They were used to study associations between a variety of dietary factors and colorectal CRC incidence. The study found that intake of fiber, calcium, and yogurt was associated with a lowered risk of CRC as there were 35 statistically significant inverse associations. These results suggest that these foods may protect against this common cancer [24]. Recently, a prospective study reported a statistically significant inverse association between greater fiber intake and the risk of CRC. A key aspect of this study was its comprehensive approach, which involved examining multiple dietary factors simultaneously and adjusting for potential confounders [35]. Another prospective study by Jun et al. (2023) in Korean adults examined the effect of dietary fiber on the causation of CRC and reported an inverse relationship between increased dietary fiber intake and the risk of gastrointestinal tract malignancies. This aligns with previous research from other regions and supports recommendations for increased fiber consumption for CRC prevention. The study also revealed that while a protective association was observed for overall dietary fiber, analysis of fiber from specific food sources (grains, vegetables, and fruits) did not yield statistically significant results, possibly because of the limited sample size and short follow-up period of the study [36].

Another systematic review and meta-analysis revealed a statistically significant positive connection between the consumption of red and processed meats and the risk of developing serrated polyps (SPs). In contrast, a fiber-rich diet was associated with a reduced risk of these polyps. However, the protective effect of fibers was primarily observed in relation to SPs and sessile serrated polyps, with no significant associations noted for hyperplastic polyps. The review also examined other dietary factors, such as vitamin D, calcium, and folate, but did not find statistically significant associations with SPs risk. The findings of this study indicate that the consumption of red meat, processed meat, and the intake of inadequate fiber may be important in the development of SPs. However, the result has some limitations. The authors found a major publication bias with respect to the association of red and processed meat with SPs risk. Further, the combined findings for fiber and folate depended on the inclusion of specific studies. The importance of these associations changed following the exclusion of certain studies. Further studies are needed to verify these results and gain insights on how they interact and affect serrated colorectal polyp development, according to these limitations [37]. A recent meta-analysis has found that eating more fiber is strongly linked to lower rates of cancer in the colon. A high-fiber diet could play a preventive role since a reduced occurrence of colorectal adenomas was observed due to an increased intake of fiber. According to the findings, following a fiber-rich dietary pattern is important and will have substantial positive public health implications [38].

**Figure 1.**
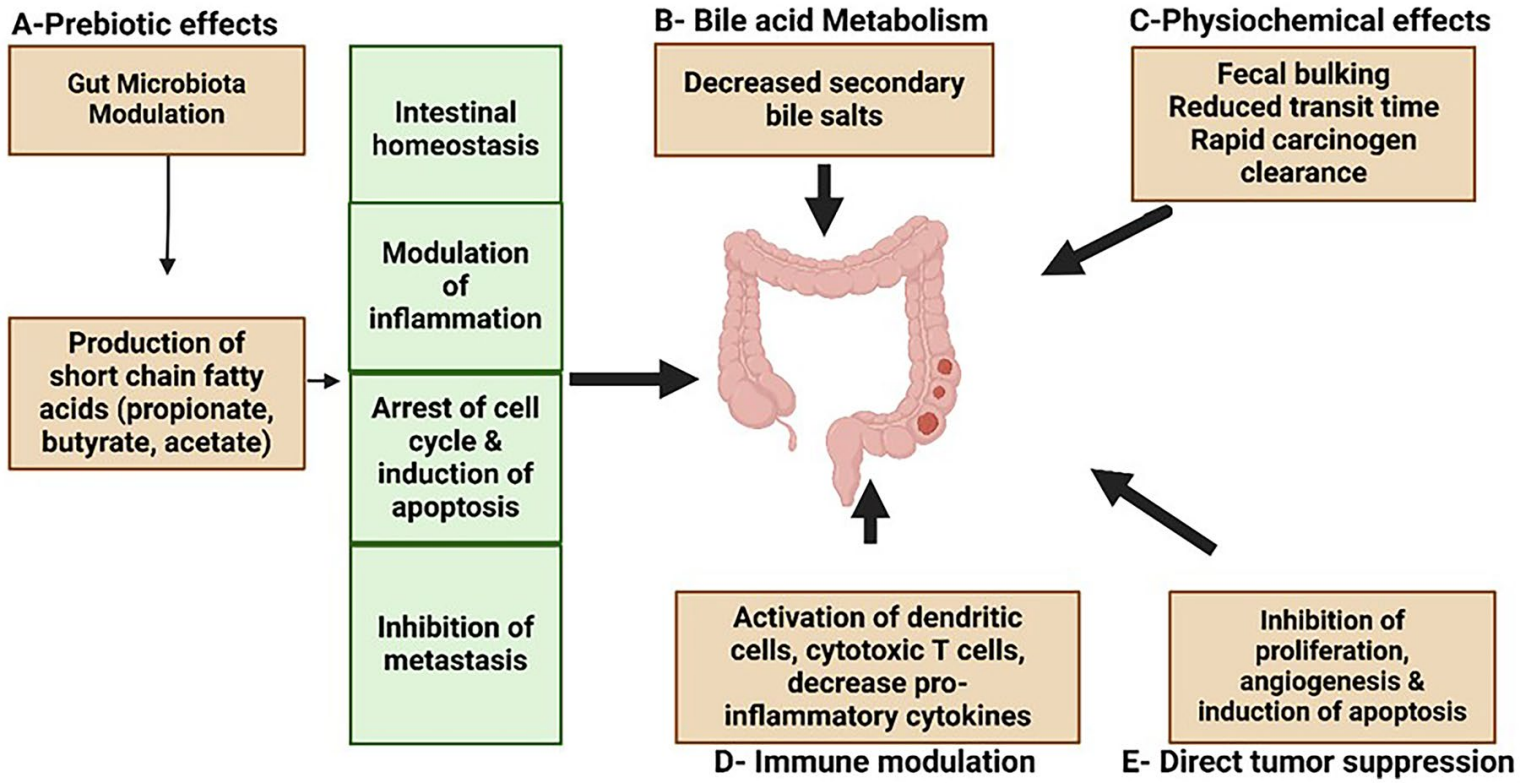
The mechanism of dietary fiber in colorectal carcinoma prevention and treatment.

A major prospective cohort study that was part of the prostate, lung, colorectal, and ovarian (PLCO) cancer screening trial investigated the link between dietary fiber intake and the risk of colorectal adenomas and cancer. The study didn’t use any specific intervention or a regular control group but assessed the self-reported fiber intake and their eventual diagnoses of CRC and adenomas. The results of the study found that greater total fiber intake (especially from cereals and fruits) was associated with a significantly lower probability of having adenomas and cancer [39]. All types of epidemiological studies have their limitations. However, these studies taken together strongly support that dietary fiber is protective against CRC. Meta-analyses or combined analyses of epidemiological studies also provide compelling evidence supporting the protective role of fiber-rich foods or dietary fiber against CRC. The convincing argument for the fiber hypothesis highlighted by these studies is the consistent protective effect of dietary fiber observed across various populations with differing dietary patterns and rates of CRC (Figure 1).

A significant etiological insight into how dietary fiber may affect the risk of CRC subtypes is provided by a large prospective cohort study by Mehta et al. (2017) that was published in JAMA Oncology. This study is unique because, unlike previous research, it demonstrates a direct correlation between diet and the microbes that reside inside the tumor rather than merely examining the impact of diet on the microbes in stool samples. This offers a more detailed understanding of how nutrition may affect the development of cancer at the actual tumor site. The study combines tumor microbiome profile and prospective clinical epidemiology data to show that a healthy diet rich in vegetables, whole grains, and fiber is substantially linked to a decreased risk of *Fusobacterium nucleatum* (*F. nucleatum*)-positive CRC, but not *F. nucleatum*-negative tumors. This finding suggests a potential mechanism by which gut bacteria may influence cancer risk by demonstrating how lifestyle factors, such as diet, can interact with specific molecular characteristics of tumors. This method emphasises how crucial it is to examine environmental and lifestyle factors in addition to molecular biomarkers in order to gain a better understanding of how various disease subtypes arise. It also emphasises how crucial it is to take tumor heterogeneity into account when assessing risk factors and preventative measures. Targeted prevention and treatment approaches may be improved by the combination of lifestyle data and microbial, genetic, and immune biomarkers, which enables a more individualised understanding of disease pathogenesis [40].

The study by Ogino et al. (2011) highlights how Molecular Pathological Epidemiology (MPE) can help bridge the gap between epidemiology and molecular pathology, leading to a better understanding of therapeutic and preventive strategies. According to the study, MPE is a developing field that integrates these disciplines to better understand the complexity of CRC. Instead of treating CRC as a single disease, MPE examines the relationships between various tumor subtypes with characteristics like MSI, CIMP, chromosomal instability, and mutations in genes like KRAS or PIK3C and factors like obesity, diet, smoking, and aspirin use. The authors emphasise that this approach enhances our ability to recognise causal relationships and creates new opportunities for prevention and precision treatment. For instance, aspirin may specifically lower the incidence of tumors with PIK3CA mutations, but the effect of obesity on prognosis varies according to the tumor’s metabolic markers. In order to overcome methodological challenges and fully utilise MPE in individualised cancer treatment, the paper also emphasises the importance of large, integrated datasets and interdisciplinary collaboration [41].

Another study by Ogino et al. (2019) emphasises how various internal and external factors, including genetics, lifestyle, environmental exposures, diet, and the microbiome, interact to affect not only cancer risk but also the molecular and cellular characteristics of tumors and surrounding tissues. These factors affect multiple biological levels, including the genome, epigenome, transcriptome, proteome, and metabolome. This manifests differences even within the same type of cancer, as individual variation in exposures, known as the exposome, and host factors come into play. The authors stress the importance of MPE in revealing these complex interactions. For example, they point out how genetic variants can change the effects of environmental exposures or how diet affects the microbiota, which in turn influences immunity and tumor development. Particular attention is focused on the role of *F. nucleatum* in CRC. It is associated with tumor subtypes, immune infiltration, prognosis, and therapy response. This positions the microbiome as both a marker and a treatment influencer. Finally, the paper emphasizes the need for better methods, including digital pathology, computational tools, and teamwork across disciplines. It also acknowledges challenges like differences in data, reproducibility, and data limitations. MPE is presented as a strong framework for improving cancer prevention, diagnosis, and treatment [42].

### 3.1. Lifestyle and environmental influences on disease mechanisms

Recent studies reinforce that multiple modifiable factors such as diet, obesity, physical activity, alcohol, smoking, gut microbiome, and environmental toxins act through molecular, cellular and metabolic pathways to influence CRC risk, progression, treatment and prognosis [43,44]. CRC development is driven by genetic and epigenetic changes that affect key biological mechanisms, such as cell death pathways (like apoptosis and autophagy), and dysregulate cell signaling networks (including Wnt, BMP, MAPK, and Notch pathways) [45]. An interplay exists between human genetics and intratumoral microbiota in CRC progression, where genetic variations, like a specific single nucleotide polymorphism (SNP) in the KCNJ11 gene, can alter the composition of the intratumoral microbiota by affecting bacterial adhesion and promote CRC by promoting the presence of bacteria like *F. nucleatum*. This interaction provides new insights into CRC development and offers potential avenues for personalized diagnostic and therapeutic strategies targeting both host genetics and the tumor’s microbial environment [46,47].

**Figure 2.**
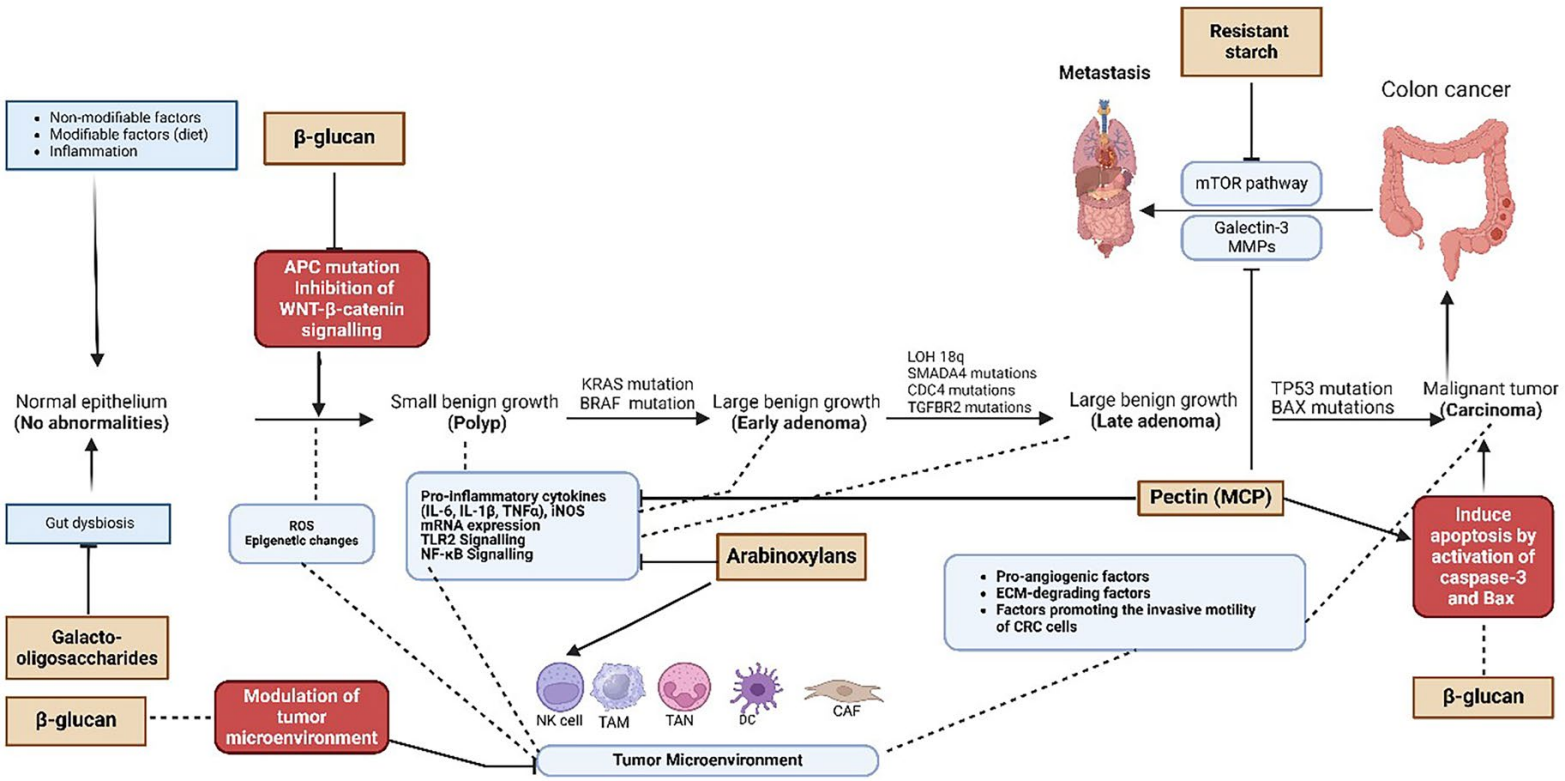
Multiple potential cellular, molecular and metabolic therapeutic targets of different types of dietary fibers in colorectal carcinoma. ROS, reactive oxygen species; ECM, extracellular matrix; CRC, colorectal cancer; MMPs, matrix metalloproteinases; NK, natural killer; TAN, tumor-associated neutrophil; iNOS, inducible nitric oxide synthase; TAM, tumor-associated macrophage; DC, dendritic cell; CAF, cancer-associated fibroblast; (⊥), an inhibitory effect is shown by the symbol.

One of the strongest influences is diet and eating habits [48]. Diets high in red and processed meat, saturated fats, and low in fiber are often connected to a higher risk of CRC. These diets can cause inflammation, oxidative stress, and create harmful substances like N-nitroso compounds and heterocyclic amines (HCAs). Heme iron found in red meat helps form N-nitroso compounds. Cooking at high temperatures creates HCAs and polycyclic aromatic hydrocarbons. Such diets can also increase bile acid secretion and disrupt gut microbiota, which can further encourage cancer development [49]. Fiber, especially from whole grains, fruits and legumes supports populations of butyrate-producing bacteria. The butyrate serves as an energy source for colonocytes, helps maintain barrier integrity, promotes apoptosis of damaged cells, and has anti-inflammatory effects [50]. Different types of dietary fibers such as β-glucan, resistant starch, pectin, arabinoxylan, galacto-oligosaccharides have distinct cellular, molecular and metabolic therapeutics targets, including modulation of Wnt/β-catenin, NF-κB, mTOR, apoptotic pathways, epigenetic regulation *via* HDAC inhibition, cell adhesion mechanisms, gut dysbiosis and microbiota-mediated signalling (Figure 2). Their combined effects result in decreased proliferation, increased apoptosis, reduced inflammation, and lowered metastatic potential of colorectal cancer cells [51]. On the other hand, low fiber/high fat diets can shift the microbiome toward dysbiosis, decrease beneficial microbes, increase pathobionts like *F. nucleatum* and *Bacteroides fragilis*, which produce genotoxins or trigger inflammatory and immune-modulating signaling [52].

Obesity is another major modifier. It contributes *via* multiple molecular mechanisms, like chronic systemic inflammation due to elevated cytokines like IL-6, TNF-α, insulin resistance and elevated insulin, which promotes cell proliferation and survival. Changes in adipokines, such as decreased adiponectin and elevated leptin and resistin, are also linked to obesity. Changes in the composition and diversity of the gut microbiota are also linked to it. Obesity and specific microbial communities can cause epigenetic remodelling (histone acetylation, methylation) in colonic epithelial cells, preparing them for oncogenic transformation, according to some recent mechanistic research in mice [53].

Physical activity appears to help offset various negative effects. It reduces systemic inflammation, improves insulin sensitivity, supports immune response regulation, and may address obesity-related dysbiosis. Studies show that physically active individuals have a lower risk of CRC, even in the presence of obesity. This likely happens because exercise affects some of these harmful molecular pathways [54]. Other factors, such as alcohol, smoking, and environmental toxins, also cause issues through DNA damage, harmful metabolites, oxidative stress, and changes in gene expression. Alcohol metabolism produces acetaldehyde, which can cause mutations. Smoking introduces numerous carcinogens, and certain environmental toxins can alter the microbiome, disrupt mucosal barrier function, lead to chronic inflammation, and promote changes in DNA methylation [55]. Every day, we are exposed to environmental pollutants like pesticides, industrial chemicals, and aflatoxins. These substances can damage our DNA and even change how our genes work, making it more likely for mutations to occur in the cells lining the colon. Exposure to these pollutants can also disrupt the immune system, making it harder for the body to recognize and remove abnormal or early cancerous cells [56].

A particularly important area of recent research is epigenetic modulation. Lifestyle and environment factors are shown to cause changes in DNA methylation, histone modifications, chromatin accessibility in colon epithelial cells. These epigenetic alterations can affect regulation of oncogenes, tumor suppressor genes, and cellular pathways such as apoptosis, proliferation, repair of DNA damage. It has been demonstrated that, in a manner dependent on the microbiota, obesity increases the activity of specific cis regulatory regions (enhancers, promoters) in the colonic epithelium (*via* histone acetylation). This indicates that the way environmental exposures result in epigenetic and transcriptional changes in the colon is altered by the presence or composition of microbes [57].

The gut microbiota, which is crucial for controlling both carcinogenic and anti-carcinogenic processes, is also impacted by lifestyle decisions. Dysbiosis, characterised by an imbalance of bacterial populations, can lead to increased production of pro-inflammatory metabolites, disruption of the mucosal barrier, and activation of carcinogenic pathways such as β-catenin and COX-2 signalling. Chronic inflammation brought on by microbial dysbiosis encourages cellular proliferation and inhibits apoptosis, fostering an environment that is conducive to neoplastic transformation. Individual differences in microbiota account for a large portion of the variation in the ways that diet, obesity, and other exposures impact the risk of CRC. For example, some people have high levels of *F. nucleatum*, which has been shown to suppress the immune system, inhibit apoptosis, and activate Wnt/β catenin signalling. The host immune milieu, including the activity of T cells, macrophages, and other immune cells, plays a decisive role in shaping the response to bacterial colonization. This immune response can determine whether colonization promotes an inflammatory and tumor-supportive microenvironment. Susceptibility is further modified by host genetic variants that affect pathways such as pattern recognition receptor signaling, DNA repair, and detoxification [58].

Lifestyle and environmental factors interact with molecular subtypes of CRC. These include microsatellite instability (MSI-high vs. MSI-stable), CpG island methylator phenotype (CIMP-high vs. CIMP-negative), and tumor location (proximal vs. distal). Evidence suggests that western diet and obesity may have stronger effects on epigenetic phenotypes in certain subtypes. Patients with MSI-high or CIMP-high tumors may also show distinct survival outcomes or treatment responses compared with those who are MSI-low or CIMP-negative. More prospective human studies are needed. However, existing data indicate that exposure-tumor subtype interactions, such as diet-CRC subtype, may explain why dietary modifications appear more protective in some populations or within specific tumor subsets [59,60].

In summary, lifestyle and environmental factors shape a complex network of cellular, molecular, and metabolic mechanisms. These include inflammation, genomic stability, immune modulation, and interactions with the microbiome. Together, they influence the initiation and progression of CRC. Targeting these factors through preventive strategies and lifestyle modifications may help reduce the burden of CRC. Such approaches also have the potential to improve therapeutic outcomes.

## 4. Preclinical evidence

Dietary fibers have garnered significant interest as beneficial food elements since the 1980s because of their significant health advantages for humans. Numerous studies have shown the beneficial effects of dietary fiber on various illnesses, including CRC. Because different types of fibers are specific bioactive compounds with diverse and distinct functional characteristics encompassing multiple diseases, we analyzed preclinical studies of different types of dietary fibers in the context of their unique role in the onset, progression and treatment of CRC and summarized the results in Table 1. Understanding the mechanisms of individual fibers offers an outline on which their therapeutic application in CRC patients may be customized.

**Table 1.** Preclinical research on how dietary fibers interact with conventional or experimental treatments for colorectal cancer.

Dietary fiber	*In vitro*	*In vivo*	Key findings	References
Oat beta-glucan		Rats given 1,2-dimethylhydrazine treatment	Reduced colonocyte proliferation, decreased the number of aberrant crypt foci and increased the activity of antioxidant enzymes	[61]
β-1,3-1,6-Glucan	HT-29 and HCT-116 CRC cell lines (human)	BALB/c mice with colon cancer cells (CT-26)	Induced apoptosis, caspase activation, cell cycle arrest, mitochondrial membrane potential disruption, and ROS generation in vitro. Suppressed tumor growth in vivo.	[62]
Oat β-glucan	Not applicable	Male Wistar rats treated with azoxymethane to induce colorectal carcinogenesis	Reduced formation of aberrant crypt foci, particularly with 3% OβGl. Increased expression of autophagy markers (LC3B-II/LC3B-I ratio, CASP-3) and apoptosis marker (cleaved CASP-3) in the colorectal mucosa, especially with 3% OβGl.	[63]
β-glucan from barley flakes (raw and roasted)	Human colon digestion model (static)	Not applicable	Increased β-glucan content after roasting, especially in thick flakes. Fermentation supernatants stimulated butyrate production. FS inhibited the growth of LT97 colon adenoma cells, with greater effects from thick flakes and longer treatments.	[64]
Resistant Starch	LoVo, HCT116, and NCM460 cell lines	AOM/DSS-treated mice and ApcMin/+ mice	Suppressed HFCS-induced colon tumorigenesis, altered gut microbiota, increased SCFAs, decreased HK2 expression and reduced glycolysis	[65]
Sugar beet pectin	HT29 and DLD1 colon cancer cells	Not specified	Weak effect on cell viability- Enhanced effect with alkali treatment- Induced apoptosis- Increased RGI to HG ratio- Decreased DE and DAc- Importance of neutral sugar side-chains (galactan and arabinan)	[66]
Modified apple polysaccharide		Mouse model of colitis-associated colon cancer (DMH/DSS-induced)	Increased apoptosis of colonic epithelial cells, reduced tumor incidence and modulated galectin-3 activity	[67]
Pectin	Dissolution testing in various pH media (simulated gastric, intestinal, and colonic fluids)	Male Sprague‒Dawley rats	Controlled release of 5-fluorouracil in the intestinal environment, improved drug release profile	[68]
Arabinoxylans (soft wheat)	Human colon cancer cells (HCT-116)	–	Reduced cancer cell viability (dose- and time-dependent)	[69]
Arabinoxylans (hard wheat)	Human colon cancer cells (HCT-116)	–	Reduced cell viability (dose- and time dependent, more effective than soft wheat AX	[69]
Galacto-oligosaccharides derived from lactulose	Not specified	Animal model (rats)	57.5% reduction in colorectal tumors Altered gut microbiota composition (increased Bifidobacterium and Bacteroides, decreased potentially harmful bacteria), reduced colorectal cancer development	[70]

ROS: reactive oxygen species; CRC: colorectal carcinoma; FS: fermentation supernatants; AOM/DSS: azoxymethane/dextran sodium sulfate; HFCS: high-fructose corn syrup; SCFAs: short-chain fatty acids; ACF: aberrant crypt foci; AX: arabinoxylans; DE: degree of esterification; RGI: ratio of rhamnogalacturonan I; HG: homogalacturonan; MCP: modified citrus pectin.

### 4.1. β-glucan

β-glucan has attracted attention in recent years as a potent immunomodulator and anticancer agent [61,71]. Nevertheless, the physical characteristics, conformations, structures, and receptor-binding affinities of β-glucans from various sources vary [72]. β-glucans constitute an integral part of the outermost layer of the cell walls of cereal grains (especially barley and oats), mushrooms, yeast, fungi, and algae. They are found primarily as β-D-glucose chains joined by β-1-3 or β-1-4 glycosidic linkages [72]. β-glucans modulate both the innate and adaptive immune systems, both directly by activating multiple immune cells and indirectly through the modulation of the gut microbiota [73]. They activate dendritic cells, macrophages, natural killer cells, and neutrophils through interactions with several key receptors, including Dectin-1 (a lectin family receptor), Toll-like receptors such as TLR2 and TLR4, and CR3 [73,74]. Dectin-1 is expressed at high levels on dendritic cells and at low levels on macrophages and neutrophils and contains an immunoreceptor tyrosine-based activation motif (ITAM), which is involved in signal transduction. The attachment of β-glucan to Dectin-1 causes the phosphorylation of tyrosine and subsequently the NF-κB pathway, which leads to the production of multiple cytokines, such as IL-6, IL-1, IL-12, IL-2, and TNF-α [75,76].

In addition, this interaction led to increased expression of B7.1 and B7.2 (costimulatory molecules) on the surface of antigen-presenting cells (APCs). These molecules bind to the T-cell receptors CD28 and CTLA-4, causing the activation and modulation of T-cell immune responses [77]. When Dectin-1 gets activated, it can also induce the secretion of cytokines from macrophages and dendritic cells together with TLR2-induced NF-κB activation [75]. These interactions also activate dendritic cells subsequently enhancing their maturation, migration and T cell antigen presenting capabilities. In general, the cytokine and costimulatory molecule secretion induces activation of Th1 responses and the generation of cytotoxic T lymphocytes [78]. Natural killer cell activation causes the release of lytic granules such as perforin and granzymes, which kill cancer cells, or they can also kill their target cells *via* receptor-mediated apoptosis [79].

In addition to its strong anticancer effects mediated through the activation of the immune system, β-glucan has the ability to attach to tumor cells directly through FAS receptors and induce apoptosis *via* the activation of caspase-3 and Bax (pro-apoptotic factors) and the inhibition of Bcl-2 (antiapoptotic factor) [80]. These diverse effects make β-glucan a promising candidate for fighting against multidrug-resistant CRC. In addition, some other pathways for inhibiting the growth of malignant cells involve β-glucans. Recently, Kim et al. (2023) demonstrated that β-glucan exhibits anticancer activity against CRC cells *via* multiple mechanisms, such as arresting the cell cycle and inhibiting tumor growth [62] (Table 1). More recently, Kopiasz et al. (2024) reported that low-molecular-weight oat β-glucan has preventive effects against CRC in rats, potentially by modulating autophagy, apoptosis, and DNA repair processes [63] (Table 1).

Additionally, to their direct effects on immune and malignant cells, β-glucans, like other dietary fibers, are well fermented by beneficial gut bacteria in the distal colon, which has various positive health consequences. Multiple SCFAs, including butyrate, acetate, and propionate, are produced by the fermentation of dietary β-glucans by a range of gut microorganisms, including *Bifidobacteria*, *Enterococcus*, and *Lactobacilli* [81]. Many studies have shown that butyrate can inhibit CRC cell proliferation by promoting the autophagy-mediated degradation of β-catenin, thereby inhibiting the Wnt/β-catenin pathway [82,83] (Figure 2). This pathway may be a potential therapeutic target for CRC because it is involved in tumor initiation, proliferation, differentiation, and metastasis of malignant cells [84]. In addition, this pathway is also linked with disabling mutations of the APC gene, which is a tumor suppressor gene linked to CRC etiology. APC-inactivating mutations are present in approximately 80% of all human colon cancers [85]. Additionally, butyrate reverses intestinal epithelial barrier failure by activating GPR109A and blocking the NF-κB and protein kinase B signaling pathways [86]. A study by Schlörmann et al. (2020) revealed that increased butyrate production from fermented β-glucan may be a key factor in inhibiting colon cancer cell growth [64] (Table 1).

The other important SCFAs are propionate and acetate, which regulate gut homeostasis by enhancing the growth of epithelial cells and promoting barrier integrity. The absence of these acids leads to intestinal imbalance and unregulated proliferation of intestinal epithelial cells, potentially contributing to CRC [87]. Overall, SCFAs play crucial roles in maintaining gut barrier integrity, gut homeostasis, induction of apoptosis, inhibition of tumor cell proliferation, and metastasis of CRC cells. Imbalances in their production due to gut microbial dysbiosis can contribute to intestinal inflammation and associated diseases. Additionally, owing to their biocompatibility, easy modification, and colon-specific degradation, β-glucans offer an exciting opportunity for colon-specific drug delivery, thus improving chemotherapy efficacy [28].

In summary, β-glucans demonstrate promising anticancer properties, especially in CRC, primarily through immune system modulation by enhancing the activity of immune cells such as macrophages, neutrophils, and natural killer cells and subsequently activating Th1 immune responses. They also exert direct antitumor effects by inhibiting cell proliferation, inducing apoptosis, and reducing tumor growth. To translate these studies into CRC clinical therapies, there is a need for RCTs that aim to develop standardized formulations and dosage regimens of β-glucan derived from different sources and explore the interaction between β-glucan and current CRC therapies to examine the synergistic or antagonistic effects as well as its long-term effects and safety profiles.

### 4.2. Resistance starch

A form of starch known as resistant starch (RS) evades digestion in the small intestine, subsequently reaching the colon, where the gut microbiota ferments it to produce SCFAs [88]. There are several types of resistant starch that are classified on the basis of their source and structure. The main type is physically resistant starch, which is found in whole wheat, oats, barley, legumes (e.g. lentils, chickpeas), and unripe bananas. The starch is physically encapsulated in the plant cell walls, making them resistant to enzymatic digestion. Another type is raw starch, which is found in raw, unprocessed starches where the granules are resistant to digestion. Raw potatoes, green bananas, and some legumes are notable examples [89].

In recent years, RS has garnered attention for its potential health benefits, particularly in the context of CRC, because of its effects on gut health, inflammation, and cancer cell proliferation. Preclinical studies have shown that, through fermentation in the colon and subsequent production of SCFAs (especially butyrate), resistant starch can influence several biological mechanisms involved in the development of CRC. These mechanisms include the induction of apoptosis, inhibition of tumor cell proliferation, modulation of inflammation, alteration of the gut microbiome, and activation of immune responses [90]. A study has shown that RS inhibits the mTOR pathway, which is involved in the proliferation, growth, and metastasis of colon tumor cells, resulting in increased survival of mice in the RS group compared with the control group [91,92]. Therefore, the mTOR signaling pathway is another potential therapeutic target for CRC prevention and treatment (Figure 2).

One of the most significant effects of RS is its ability to modify the gut microbiota. As RS reaches the colon without being digested, it serves as a substrate for fermentation by gut bacteria, leading to the production of SCFAs, primarily butyrate. Butyrate is a key SCFA that has several beneficial effects on gut health and cancer prevention. Zhang et al. (2024) demonstrated that resistant starch modifies the microbial population, leading to elevated intestinal SCFA levels and decreased glycolysis and colon carcinogenesis through the downregulation of HK2 [65] (Table 1). Because there is strong evidence both *in vitro* and *in vivo* that RS is a functional food beneficial to the colon, a prospective therapeutic approach that targets RS to counteract the negative effects of high-fructose corn syrup (HFCS) could be effectively employed. According to Hu et al. (2016), resistant fibers may protect against CRC linked to colitis. This protective effect is linked to notable changes in the gut microbiota, the synthesis of SCFAs, elevated GPR43 expression, and decreased expression of inflammatory cytokines and cell proliferation [93] (Table 1). In summary, the key anticancer effects of resistant starch include inhibition of tumor cell proliferation, promotion of apoptosis, inhibition of metastasis, modulation of inflammation, alteration of the gut microbiome, and activation of immune responses.

### 4.3. Pectin

Pectin is a naturally occurring, soluble gelatinous polysaccharide that occurs in the cell walls and middle lamella of plants. It is especially abundant in fruits, where it serves as a structural component that helps to hold cells together. It consists of galacturonic acid residues, which are connected by α-1,4 glycosidic bonds [94]. It is widely utilized commercially for its stabilizing and gelling properties, particularly in the production of jams and jellies, and as a stabilizer in fruit-based products. Furthermore, its thickening and emulsifying properties contribute to the texture of a variety of food items [95]. Lemons, oranges, grapefruit, and lime contain pectin, especially the peel and the membranes. Citrus pectin is used in many commercial food products and supplements. Apples are another great source of pectin, especially in their peels and cores. Pectin from apples is frequently used in foods and as a dietary supplement [96]. Strawberries, blackberries and raspberries contain lesser amount of pectin mainly present in fruit pulp. Pears and apples are rich in pectin but mainly when they are not ripe [96,97].

Numerous preclinical studies have investigated the potential therapeutic effects of pectin in the treatment and prevention of CRC [98,99]. Pectin and its derivatives, such as modified citrus pectin (MCP), have shown promise in various experimental models because of their effects on tumor growth, metastasis, immune modulation, and the gut microbiota [66]. MCP refers to a modified version of pectin that is easier to absorb and that is more bioavailable and therapeutic. MCP binds galectin-3 which is probably one of the main mechanisms through which it exerts anticancer effects. Galectin-3 is involved in the growth, adhesion, and metastasis of cancer cells. By inhibiting galectin-3, pectin can reduce tumor growth, suppress metastasis and inhibit tumor cell proliferation [67] (Figure 2). Research on pectin has shown its ability to restrain cancerous cells from growing by blocking the cell cycle at the G1 phase, allowing it to not proceed to mitosis and further divided [100,101]. Pectin and MCP help prevent malignant cells from attaching to the blood vessels and the tissues around them, reducing their movement into other organs and tissues. Research indicates that pectin may prevent tumor cell invasion by blocking matrix metalloproteinases (MMPs), which damage the extracellular matrix (ECM) [102].

Several studies demonstrated that pectin could inhibit MMPs. MMPs are enzymes that disrupt ECM and facilitate tumor cell invasion. By reducing MMPs activity, pectin can decrease the ability of malignant cells to invade surrounding tissues and metastasize [103]. Additionally, pectin, particularly MCP, has been shown to decrease the levels of proinflammatory cytokines such as IL-6, TNF-α and IL-1β, which are often elevated in the tumor microenvironment and contribute to tumor progression [104]. Moreover, pectin can modulate the activity of the NF-κB pathway, which plays a key role in the expression of multiple genes involved in angiogenesis, cell proliferation, inflammation, apoptosis, and the development of CRC and its metastases [98,105,106]. Therefore, the NF-κB pathway may be a potential target for therapeutic intervention aimed at inhibiting angiogenesis and further reducing the progression of CRC (Figure 2).

Elyagoby et al. (2013) developed a colon-specific drug delivery system for 5-fluorouracil (5-FU) by combining pectin, zinc and ethyl cellulose. The preferential release of 5-FU under simulated colonic conditions was revealed in *in vitro* studies while the targeted delivery to the colon was confirmed *in vivo* in Sprague-Dawley rats. The system achieved improved drug delivery and delayed release through the formation of an ethyl cellulose plug within the capsule. The study concluded that this approach can enhance colon cancer treatment by maximizing drug efficacy at the target site and minimizing systemic exposure [68].

### 4.4. Arabinoxylans

Arabinoxylans (AXs) are a type of hemicellulose, a complex polysaccharide found in the cell walls of various cereals, such as wheat, barley, oat, corn, rye, and rice [107]. They are primarily composed of a backbone of xylose units, with branches of arabinose attached to the xylose residues [108]. Extensive *in vitro*, animal, and human studies clearly show that arabinoxylan derived from cereals has all the characteristics of prebiotics. These include resistance to breakdown in the intestinal tract, fermentation by probiotics, and the selective promotion of beneficial gut bacteria, especially *Bifidobacterium* and *Lactobacillus* bacteria [109]. Recently, the biological pathways of rice bran arabinoxylan compound (RBAC) and its effects on cancer treatment were analyzed in a review article. The conclusions of these 38 articles indicate that arabinoxylan promotes apoptosis and restores immune function, enhancing inflammatory responses and blocking tumorigenesis. It works synergistically with chemotherapeutic agents and has improved the treatment response of patients with liver cancer, including reduced recurrence and prolonged survival. Additionally, arabinoxylan protects against oxidative stress and decreases the adverse effects of chemotherapy. A study revealed that arabinoxylan therapy increased the survival odds of CRC patients by 4.02 times in the first year and 2.89 times in the second year. Overall, RBAC has significant potential in cancer treatment [110]. Biobran (RBAC) stimulates type 1 effector cells when it is given at 10 to 45 mg/kg [111]. A previous study analyzed arabinoxylan from two different wheat genotypes (soft and hard) and reported that both forms decreased the viability of a colon cancer cell line (HCT-116); however, hard wheat arabinoxylan was more effective than soft wheat AXs [69] (Table 1).

A study performed by Ooi et al. (2023) revealed that rice bran arabinoxylan compounds influence both innate and adaptive immune responses. RBAC enhances NKC activity by increasing NKC cytotoxicity and IFN-γ production without affecting the NKC count. This effect is often dose dependent and can be further enhanced by IL-2 [112]. Clinical trials support these preclinical findings, suggesting that RBAC may be beneficial for increasing NKC activity in humans, particularly in older adults, and may promote dendritic Cell (DC) maturation. DCs process antigens and present them to T cells, activating the adaptive immune system. As a result of DC maturation, RBAC indirectly enhances the proliferation of both B and T lymphocytes [113].

In summary, cereal-derived AXs may significantly contribute to the onset, progression, prevention and treatment of CRC. They promote gut health by acting as prebiotics that enhance beneficial bacteria and increase the production of SCFAs such as butyrate, which have antitumor and anti-inflammatory properties. AXs also help reduce inflammation, improve the gut transit time, and offer antioxidant protection against oxidative stress. While not a primary treatment, AXs can enhance the immune response as an adjuvant therapy by stimulating natural killer cells and macrophages, as well as promoting interleukin-2 production.

### 4.5. Galacto-oligosaccharides

Galacto-oligosaccharides (GOSs) are a group of soluble nondigestible carbohydrates found in various raw and processed foods that act as fermentable substrates for the gut microbiota. They are present in human breast milk, dairy products, legumes such as beans and lentils, and root vegetables such as garlic and onion. Fermented foods such as yogurt, kimchi, kefir, and sauerkraut also contain GOSs [114]. GOSs may play a protective role against CRC through several mechanisms. One of their potential targets is gut dysbiosis. Dysbiosis, characterised by an imbalance of bacterial populations, can lead to increased production of pro-inflammatory metabolites, disruption of the mucosal barrier, and activation of carcinogenic pathways such as β-catenin and COX-2 signalling. Chronic inflammation brought on by microbial dysbiosis encourages cellular proliferation and inhibits apoptosis, fostering an environment that is conducive to neoplastic transformation. GOSs act as prebiotics because they selectively increase the growth of beneficial gut microbiota that produce SCFAs. SCFAs induce apoptosis and inhibit inflammation and tumor cell proliferation in colon cancer cells [70]. By fostering a healthy gut microbiome and increasing gut barrier function, the GOSs can help decrease the risk of inflammation and maintain a balanced gut environment, which is essential for colorectal health [115]. Chronic inflammation is an established risk factor for CRC, and the GOSs can help modulate immune responses and decrease inflammation in the gut, contributing to a lower cancer risk [70]. While GOSs are not the primary treatment for CRC, they may serve as effective adjuvant therapies. By enhancing immune function and improving the gut response to treatment, GOSs can support the effectiveness of conventional cancer therapies and reduce treatment side effects. They may also improve the overall well-being of patients undergoing cancer treatment by promoting gut health and mitigating gastrointestinal symptoms [116]. Overall, while more research is necessary to establish definitive roles and mechanisms, GOSs shows promise as a functional ingredient in diet-based strategies for CRC prevention and as supportive therapy during treatment.

In summary, preclinical studies provide strong foundational evidence supporting the role of dietary fiber in CRC prevention and treatment. These studies, which were conducted using *in vitro* models (such as CRC cell lines) and *in vivo* models (such as mice or rats), identified several key mechanisms (common and distinct) by which dietary fiber can influence CRC development, progression, and treatment. Dietary fibers are fermented by the colonic microbiota, but their site and degree of fermentation differ; for example, low-molecular-weight dietary fibers, such as oligosaccharides and inulin, are rapidly fermented by gut bacteria in the proximal colon, selectively promoting the growth of probiotic bacteria such as *Bifidobacteria* and *Lactobacilli*; therefore, they are known as prebiotics. On the other hand, fermentation of high-molecular-weight fibers, such as β-glucan, resistant starch, pectin, and arabinoxylan, occurs at a slow rate and not exclusively in the proximal colon. Because of dietary fiber fermentation, SCFAs are produced, which maintain intestinal homeostasis, suppress tumor growth by promoting apoptosis, arrest the cell cycle, reduce inflammation, and inhibit metastasis (Figure 1). They also positively alter the gut microbial composition, which is essential for maintaining a healthy gut environment and preventing tumorigenesis. These findings confirm the vital role of dietary fibers in enhancing gut health, which is closely linked to CRC prevention and management.

Since each type of fiber has unique biological and structural characteristics, they also have distinct but complementary effects on CRC progression and treatment. For example, β-glucan has significant antitumor effects by inducing apoptosis in CRC cells through the activation of caspase pathways, suppressing tumor-promoting pathways, including the Wnt/β-catenin signaling pathway, and reducing tumor cell proliferation. These results suggest that β-glucans directly target cancer cells and disrupt their growth and survival mechanisms. Moreover, β-glucan can activate both the innate and adaptive immune systems directly by attaching different receptors to immune cells, enhancing T-cell infiltration in tumor tissues, and modulating cytokine production to reduce inflammation and promote antitumor immunity (Figure 2).

Resistant starch inhibits mTOR and downregulates HK2 pathways, which are involved in the proliferation, growth, and metastasis of colon tumor cells. Pectin inhibits galectin-3, a protein that plays a crucial role in cancer cell growth, adhesion, and metastasis, and inhibits matrix metalloproteinases, enzymes that disrupt the ECM and facilitate tumor cell invasion. Pectin can also modulate the activity of the NF-κB pathway. Arabinoxylans enhance NKC activity by increasing NKC cytotoxicity and IFN-γ production without affecting the NKC count. This effect is often dose-dependent and can be further enhanced by IL-2. Galacto-oligosaccharides modulate gut dysbiosis by promoting the growth of probiotic bacteria such as lactic acid and bifidobacteria and changing the overall composition of the gut microbiota.

Additionally, fiber-like pectin has been shown to enhance the efficacy of 5-fluorouracil (5-FU), thereby increasing cancer cell death and reducing side effects. This highlights the potential of dietary fiber as an adjunct to improve the outcomes of standard cancer treatments. Finally, preclinical studies on fibers such as psyllium husk, arabinoxylan, and acacia gum have demonstrated that these fibers improve intestinal barrier function by enhancing gut integrity, thereby reducing DNA damage and preventing oxidative stress. They also decrease the levels of inflammatory markers and tumor-promoting factors in the colonic environment. These findings emphasize the translational potential of dietary fiber, paving the way for clinical trials to explore its use in CRC prevention and therapy. However, while these results are promising, the translation of findings from animal models and cell lines to humans requires careful validation in clinical settings. Future research should focus on optimizing fiber types, combinations, and doses to maximize therapeutic benefits in CRC patients.

## 5. Clinical evidence

We have included several high-quality peer-reviewed clinical studies/trials that have investigated the role of diverse types or kinds of dietary fiber in CRC prevention and as adjuvant therapy. Key aspects of these studies, such as the type of study, total number of subjects enrolled in the study, intervention type, and main findings of the study, are provided in Table 2. A pilot study by Okuno and Uno (2011) examined the potential of Lentinula edodes mycelia (LEM) extract to alleviate chemotherapy-related side effects in eight patients with advanced gastric cancer or CRC. The extract has various polysaccharides, including β-glucans, which are known for their immunomodulatory and potential antitumor properties. Patients received 1800 mg/day LEM orally during their second 4-week course of chemotherapy, and their first course was used as a baseline comparison. The results indicated improvements in quality of life measures, particularly nausea, appetite loss, and fatigue, suggesting that LEM may help reduce chemotherapy-related side effects [117]. Nevertheless, the study lacked a true control group and had a small sample size, there was the subjective assessment of quality of life and limited exploration of cellular mechanisms, which severely limit the ability to generalize these findings. There is a need for larger and better studies to confirm that LEM is effective and safe for CRC patients.

**Table 2.** Clinical studies investigating the effects of dietary fiber as preventive and adjuvant therapy in CRC patients.

Study Type/Subjects	Intervention	Control Group	Main Outcome	Reference
A pilot study, 8 patients with advanced GIT cancer (gastric or colorectal) undergoing chemotherapy	LEM extract 1800 mg/day orally during the second course of chemotherapy (4 weeks)	The first chemotherapy course without LEM served as a baseline comparison for each patient.	Improvement in quality-of-life scores related to chemotherapy side effects, particularly nausea, appetite loss, and general fatigue.	[117]
Double-blind, randomized, crossover study with 21 participants who were healthy human volunteers	Red meat diet with HAMSB supplement	Red meat diet alone	Consumption of HAMSB with a red meat diet decreased the levels of O6MeG adducts in rectal tissue, a biomarker of DNA damage associated with CRC risk. Alteration in gut microbiome composition was also observed.	[118]
23 healthy volunteers in a randomized crossover design	Four 4-week dietary interventions: A HRM diet (300 g/day lean red meat) and an HRM diet that includes butyrylated high amylose maize starch as supplement. HRM with 40 g/day butyrylated high amylose maize starch.	The study employed a crossover design where participants served as their own controls, undergoing all dietary interventions. The initial “entry diet” and subsequent “washout” periods likely represent control phases for comparison. Each group’s usual diet served as the control.	Alterations in the amounts of oncogenic mature miRNAs in the tissue of the rectal mucosa, such as miR21 and miR17-92 cluster miRNAs were observed.	[119]
Dietary intervention study; 20 African Americans and 20 rural South Africans	Two-week food exchange: African Americans consumed a LFHF African-style diet; rural Africans consumed a HFLF Western-style diet.	Each group’s usual diet served as the control.	In African Americans, LFHF increased butyrate production, reduced mucosal proliferation and inflammation, altered gut microbiome composition toward fiber fermenters, and decreased secondary bile acid concentrations. Rural Africans on the Western diet showed opposite effects.	[120]
Randomized, controlled trial, 80 patients with advanced CRC	Oral administration of superfine (β-1,3-Glucan) derived from Lentinus edodes, commonly known as the shiitake mushroom SDL for 12 weeks	Standard chemotherapy regimen without SDL	No significant difference in overall survival between SDL and control groups. SDL improved quality of life scores in some patients, particularly those with initially low QOL scores. Patients with higher lentinan-binding PBMs tended to have greater QOL improvements. Primary Outcome: Change in rectal polyp number after one year. There is no statistically significant difference between the three groups. Secondary Outcomes: Polyp size, patient compliance, and dietary intake. A more pronounced effect from the high-fiber supplement during the middle 2 years of the 4-year trial, particularly for those consuming >11 g/day of fiber. Vitamins C and E may have had a weak independent effect.	[121]
Double-blind, randomized, placebo-controlled study. Fifty-eight patients have familial adenomatous polyposis	Three treatment arms: 1. High wheat fiber supplement (22.5 g/day providing 18 g of dietary fiber). 2. Vitamins C (4 g/day) and E (400 mg/day). 3. Combination of fiber and vitamins.	Placebo for both fiber and vitamins	Primary Outcome: Change in rectal polyp number after one year. There is no statistically significant difference between the three groups. Secondary Outcomes: Polyp size, patient compliance, and dietary intake. A more pronounced effect from the high-fiber supplement during the middle 2 years of the 4-year trial, particularly for those consuming >11 g/day of fiber. Vitamins C and E may have had a weak independent effect.	[122]
Randomized controlled trial. A total of 1303 patients completed the study, with 719 in the high-fiber group and 584 in the low-fiber group. The study duration was approximately 3 yrs.	Subjects were randomly assigned to receive either a high-fiber supplement (13.5 g of wheat-bran fiber per day) or a low-fiber supplement (2 g of wheat-bran fiber per day)		Wheat-bran fiber supplements did not offer any protection against recurrent colorectal adenomas.	[123]
Randomized controlled trial; 201 patients who underwent polypectomy for adenomatous colorectal polyps	Counseling on a LFHF: <50 g/day or 20% of total energy in-take (whichever was less) 50 g fiber/day	Usual diet (high fat, low fiber, typical Canadian diet)	Absence of statistically notable difference in the cumulative incidence of neoplastic polyp recurrence between the LFHF and control groups after 2 years of follow-up. However, exploratory analysis suggested a reduced risk in women and an increased risk in men in the LFHF group.	[124]
Double-blind, randomized, placebo-controlled study. 825 patients with at least two colorectal adenomas	Wheat bran, Lactoba-cillus casei, or a combination of both	Placebo	Absence of notable difference in the development of new colorectal adenomas among the interven-tion groups compared to the placebo group after 3 years of follow-up.	[125]
Double-blind, randomized, placebo-controlled trial, Patients with resected adenomatous colon polyps	Wheat bran fiber and calcium supplementation	Placebo	Absence of significant difference in fecal bile acid levels between the intervention and control groups after nine months of supplementation.	[126]
Randomized, double-blinded (partially), placebo-controlled trial, Patients with at least one resected colorectal adenoma	Reduced dietary fat (25% of total calories), wheat bran fiber (25 g daily), and β-carotene supplementation (20 mg daily)	Usual diet	Absence of significant effect of the interventions on the incidence of new adenomas.	[127]
Randomized controlled trial. Individuals who have had colorectal adenomas recently	LFHF diet (20% calories from fat, 18 g fiber/1000 kcal)	Usual diet	Absence of notable difference in adenoma recurrence rates between the intervention and control groups after four years of follow-up.	[128]

LFHF: low-fat high-fiber diet; HFLF: high-fat low-fiber; NK: natural killer; QOL: quality of life; HRM: high-red meat; HAMSB: butyrylated high-amylose maize starch; LEM: lentinula edodes mycelia; SDL: superfine dispersed lentinan; PBMs: peripheral blood monocytes.

A double-blind, randomized, and crossover study in healthy human subjects has shown that a red meat-rich diet significantly elevates O6-methyl-2′ deoxyguanosine (O6MeG) in the human colonic biopsies. O6MeG shows DNA damage and has a link with risk of CRC. Adding high-amylose maize starch butyrate to a high-red meat diet prevented O6MeG adducts from increasing. According to study, HAMSB can prevent damage done to the DNA in the rectal tissue by red meat [118]. The gut microbiota composition was altered in both the red meat diet and the red meat plus HAMSB diet, but these changes could not entirely explain the observed decrease in O6MeG adducts due to HAMSB. So, the gut microbiota’s function in this mechanism needs to be explored further.

A crossover randomized trial assessed the effects of red meat with resistant starch consumption on miRNA expression in 23 healthy subjects. Research shows that oncogenic miRNA expressed in rectal mucosa is influenced by the consumption of red meat as it leads to increased levels of miR-21 and miR-17-92 which promotes cell proliferation of the rectal mucosa. Adding resistant starch to red meat intake reduced the effects of red meat on microRNA expression, particularly that of the microRNA-17-92 family. This means resistant starch can prevent the side effects of a high red meat diet [119]. This study has a number of limitations, including a small sample size (23 subjects) and a high variability in mRNA levels which made it difficult to arrive at any definitive conclusion. The results were mostly shown correlations and not causal between diet and expression of miRNA. The period in between the dietary interventions may have been less as noted by the persistent increase in the cell proliferation. Besides, the current analysis does not include other food parameters, and it was limited to red meat and resistant starch. In the end, the study looked at cell proliferation but not development of aberrant crypt foci regarded as precancerous lesions. The observed changes could not be directly linked to a risk of CRC.

In a study by O’Keefe et al. (2015), researchers compared rural Africans and African Americans with respect to diet-related differences in gut microbiome and biomarkers associated with CRC. The study’s crossover design allowed each group to serve as its own control by switching to the other group’s typical diet for two weeks. People from rural Africa who eat a traditional diet that is high in fiber and low in fat were switched to a Westernized diet. This Westernized diet was deficient in fiber and had a lot of fat. African Americans often eat a Western diet deficient in fiber and high in fats. The researchers noticed important changes in the gut bacteria and butyrate levels within both groups and in both cases. When rural Africans ate a Western-style diet, their gut butyrate levels decreased significantly, while their secondary bile acid, p-cresol, and fecal pH all increased. When African Americans ate the African-style diet high in fiber, their levels of butyrate increased, and cancer-promoting markers decreased. The changes were mostly due to changes in gut microbiome composition. The study pointed out that a typical rural African diet which is fiber-rich and low in fat promotes a gut environment that reduces colon cancer risk. People are consuming western foods in high amounts which are deficient in fiber and high in fat. This may disrupt this protective environment and increase cancer risk. Researchers found that a total daily fiber intake higher than 50 g may be critical for public health in the prevention of colon cancer, and particularly in populations with a high basal state of chronic inflammation [120].

A pilot study by Hazama et al. (2009) investigated the efficacy and safety of orally administered superfine dispersed lentinan (SDL), a beta-glucan from Shiitake mushrooms, in 80 patients with metastatic CRC. This was a pilot study, not a randomized controlled trial, so there was no distinct control group. Patients received SDL orally alongside their ongoing chemotherapy regimen. This study revealed that SDL administration improved several aspects of QOL, including appetite loss, fatigue, and pain, and enhanced immune function in some patients. These findings indicate that lentinan binds to peripheral blood monocytes. The mode of action of lentinan involves T-cell-dependent immune activation facilitated by monocytes and macrophages. While the study does not explicitly state that lentinan directly binds to T cells, it suggests its involvement in the downstream immune response. Other studies have suggested that lentinan can bind to receptors such as toll-like receptor 2, CD11b, and dectin-1, which are found on macrophages and monocytes [129]. Another study suggested a binding site on human leukocytes similar to the C3b receptor [130]. While the study did not focus primarily on survival, it noted a potential trend toward improved survival in patients receiving SDL. The authors concluded that oral SDL is safe and may improve QOL and immune function in advanced CRC patients receiving chemotherapy, warranting further investigation in larger, controlled trials [121]. This study has various limitations, including a limited sample size (80 patients), a heterogeneous patient population, and a lack of detailed information on the chemotherapy regimens used. Subjective quality of life assessment, a focus on short-term outcomes (12 weeks), and an incomplete understanding of the mechanism of action of SDL further limit the study. Despite these limitations, the study suggests the potential benefits and safety of SDL, warranting further investigations with larger, more rigorous studies.

Another RCT investigated the role of wheat fiber supplementation, with and without supplemental vitamins E and C, in the development of rectal polyps in individuals with familial adenomatous polyposis (FAP). Compared with the control group, the high-fiber group presented a statistically significant reduction in the number of polyps, whereas the vitamin group presented a nonsignificant trend toward fewer polyps. Researchers have concluded that high-fiber wheat bran supplements can effectively suppress rectal polyp formation in patients with FAP, potentially offering a noninvasive strategy for managing this precancerous condition [122]. The limitations of the RCT include a small sample size (58 completing the 4-year trial out of 72), focusing on a specific population (familial adenomatous polyposis), challenges in patient follow-up leading to missed examinations, and lack of detailed histological assessment of polyps. These factors limit statistical power, generalizability, and ability to determine the specific intervention effects on polyp morphology or progression.

Alberts et al. (2000) conducted an RCT investigating the effect of high-fiber cereal intake on the reappearance of colorectal adenomas (CRAs). The participants had previously undergone colonoscopy and had their adenomas removed. The intervention group was given a daily supplement of fiber-rich cereal, whereas the control group received a fiber-deficient cereal supplement. This study revealed no notable difference in adenoma recurrence rates between the fiber-rich and fiber-deficient groups, indicating that adding wheat bran fiber to the diet, as applied in this study, did not inhibit the recurrence of CRAs [123]. The authors of this RCT faced great challenges in recruitment and compliance. In fact, only about one-third of the eligible subjects completed this trial. Even those who successfully completed the trial, adherence to taking the daily cereal supplement, became progressively poor in all groups, especially poor in the fiber-rich group. This means that if we didn’t see any difference, the amount of fiber might be too low, or the period of time would not be enough. Also, because the study population had relatively high baseline fiber intakes, it is possible that it would have been hard to detect any benefit. The follow-up duration of three years may not have been long enough to evaluate the effects of high-fiber supplementation on adenoma’s recurrence.

McKeown-Eyssen et al. (1994), conducted a randomized controlled trial that evaluated the role of a low-fat high-fiber diet (LFHF) on recurrent polyps of the colon. The trial was conducted on 201 individuals with a history of polyps. The intervention group was advised to follow an LFHF diet; the control group was given advice to follow their normal diet, with no specific dietary advice. The researchers wanted to know how many new cancer-causing polyps developed in each group after 3 years. The result of this study shows no statistically significant difference in polyp recurrence rates in the LFHF and control groups. The researchers found out that LFHF probably does not reduce the chance of getting colorectal polyps again [124]. The current study has many limitations such as compliance with the recommended LFHF diet was not ideal and could therefore weaken the effect of the intervention. The study used one’s dietary self-reports which are prone to recall bias or inaccuracies. The fact that follow up was only done for a short period of 3 years can also be a reason why the influence of dietary changes on polyp recurrence did not get captured. The study group included mostly white, middle-class people, so the results may not apply to other people.

Another randomized controlled trial examined the preventive impact of wheat bran and/or *Lactobacillus casei* (*L. casei*) administration on colorectal tumors in patients with a history of CRC. The trial included 398 participants who were allocated to one of four groups: wheat bran, *L. casei*, wheat bran + *L. casei*, or a control group receiving neither intervention. The main outcome, as determined by colonoscopy, was the recurrence of new colorectal tumors within three years. The incidence of new colorectal tumors in the four groups did not differ significantly according to the study. Therefore, the authors concluded that neither wheat bran fiber nor *L. casei* administration, alone or in combination, effectively prevents the growth of new colorectal tumors in this high-risk population [125]. This research has multiple significant limitations that need to be recognized. This research was not conducted in a double-blinded manner, indicating that both the participants and the researchers knew the group assignments, which might have led to bias. The authors claim this is unlikely to have had much impact on results. Still, it could have been a potential confounding factor. Moreover, as the wheat bran fiber and *L. casei* probiotic used for dietary supplementation in the study were selective, the results may not necessarily apply to other types of fiber and/or probiotics. While the follow-up period of 3 years is considered long for this type of study, longer follow-up might have different effects.

Alberts et al. (1996) conducted an RCT to assess whether the provision of calcium and wheat bran fiber supplements post-surgery for adenomatous colon polyps altered bile acid levels. In this trial involving fifty-eight patients, the patients were randomized into one of four different treatment groups, either wheat bran fiber or calcium carbonate, a combination of wheat bran fiber and calcium carbonate or placebo. Fecal bile acid levels and excretion rates were the primary outcomes measures. The results showed that neither treatment with wheat bran fiber, calcium alone nor in combination significantly differed the fecal bile acid levels as compared to those on placebo [126]. This study used a low dose of 2 g/day and a high dose of 13.5 g/day wheat bran fiber, which is quite low compared with the current recommendations for dietary fiber intake, which range from 25 to 30 g/day for adults. The higher dose used in the study (13.5 g/day) represents a substantial portion of this recommended intake, but it is important to remember that this was supplemental fiber, in addition to whatever fiber participants were already consuming in their usual diets. The lower dose (2 g/day) is considerably less than the recommended intake. Whether these doses were sufficient to exert a meaningful effect on bile acid levels is a question the study itself sought to address, and the results suggest they were not. Notably, different kinds of fibers have different physiological effects, and this study focused specifically on wheat bran fibers. Finally, the study focused on fecal bile acids, and while this is considered an important marker, it may not fully reflect the complex interplay of factors influencing colon cancer risk.

MacLennan et al. (1995) conducted a randomized controlled trial looking at whether dietary changes could impact the reappearance of CRAs. A group of 424 patients with an established history of adenomas was studied to prevent recurrence of adenomas in this high-risk group. The subjects were randomly assigned to one of eight different groups to assess the impact of the low-fat diet, wheat bran fiber, and beta-carotene. Combination of the above strategies in multiple fashions (alone or together) was tested. The control group did not receive any dietary intervention. New adenomas formed after one year as detected by colonoscopy were the main outcome. The interventions alone or in combination did not significantly reduce adenoma recurrence in the study. The study concluded that these dietary changes, at the levels tested, don’t seem to prevent the recurrence of adenomas [127]. There are several limitations related to this study, such as that the dietary interventions were poorly followed, which may have reduced the observed effects. The occurrence of new adenomas was lower than expected, which may have limited the study’s ability to detect statistically significant differences. Moreover, even though the participants were randomly assigned, there were some differences in the baseline between the groups. However, the authors claimed that these differences were small and unlikely to impact the results greatly. In the end, 12 months may not be enough to understand the true impact of changing diet on the recurrence of adenomas. Considering the limitations, it is not as straightforward to study dietary intervention and cancer risk. Although the interventions were not significant, limitations of study suggest offerings of further research involving larger sample sizes, longer follow-up periods and better assessment of compliance with dietary interventions are needed to determine the definite role of dietary factors on CRAs recurrence.

An RCT by Schatzkin et al. (2000) evaluated effect of low fat and high fiber diet on CRAs reappearance. There were 2079 subjects who had CRAs in the study. The intervention group was told to eat a diet that was low in fat, rich in fiber, fruits and vegetables whereas there was no advice for the control group. Recurrence of adenomas after four years was considered the primary outcome and was assessed by colonoscopy. This study revealed no notable difference in the rates of adenoma recurrence between the intervention and control groups [128]. This study has several limitations: while the study was randomized, it was not possible to blind participants or endoscopists to dietary intervention, potentially introducing bias. Additionally, although over 90% of the participants completed the study, the possibility of loss to follow-up influencing the results could not be entirely ruled out.

A randomized phase II trial by Limburg et al. (2011) investigated the impact of sulindac, atorvastatin, and a prebiotic fiber supplement (ORAFTI^®^ Synergy1, primarily inulin) on the chemoprevention of CRC. The study concluded that none of the interventions, alone or in combination, significantly reduced the number of rectal aberrant crypt foci (CRC risk biomarkers). The interventions and the control had similar effects on mucosal proliferation and apoptosis, suggesting that there was no significant effect on the cellular processes relevant to cancer development. The study may not have been able to detect small but potentially significant effects due to the relatively small sample size [131].

More recently a RCT by Stene et al. (2025) published in Clinical Nutrition found that synbiotic interventions reduced radiation-induced tissue damage in patients with rectal cancer by modulating the mucosal microbiota and decreasing local inflammation. The protective effects were primarily localized to the rectal tissue. The study involved 30 patients with rectal adenocarcinoma undergoing preoperative radiotherapy, divided into a control group, a fiber group, and a synbiotics group. The study collected samples to measure various factors including inflammatory markers and microbiota composition. Analysis showed lower inflammation and fibrosis in the synbiotics group’s rectal mucosa. Both fiber and synbiotics groups have a reduction in white blood cell counts post-radiotherapy. The study concluded that short-term synbiotics treatment can reduce radiation damage to the rectal mucosa [132].

In a recent randomized controlled trial by Ji et al. (2025), patients after CRC surgery were examined on the benefits of dietary fiber. A total of 164 patients were enrolled in a controlled trial. The results indicated that the enteral diet formula with enriched dietary fiber improved inflammation. The nutritional and immunity markers are all better than units supplemented with traditional nutritional mixes. Additionally, the beneficial effects of fiber were proficiently anticipated using machine learning models, which identified four key physiological markers: procalcitonin, prealbumin, albumin, and interleukin-1. In summary, the recent trial demonstrated that early dietary support with fiber may promote a quicker recovery and better control of inflammation after surgery. Finally, the study illustrated how machine learning could be used to create individualised treatment regimens [133].

In summary, the findings of clinical trials are inconclusive; some clinical studies have demonstrated a positive impact of dietary fiber intervention in preventing CRC, such as a double-blind, randomized crossover trial by Leu et al. (2015) in healthy human participants, which revealed that high-amylose maize starch butyrate may have a protective role against red meat-induced DNA damage in the rectum, thus decreasing the risk of CRC [118]. This trial also revealed that wheat fiber supplementation suppressed rectal polyp formation in familial adenomatous polyposis patients [122]. Another pilot study revealed that beta-glucan reduced chemo-related side effects and activated immune cells, thus improving the quality of life of patients [117]. Furthermore, a randomized crossover trial showed that adding butyrylated high-amylose maize starch to a diet high in red meat partially reversed these effects, especially for miR-17-92. These findings suggest that resistant starch may have a protective role against the negative effects of high red meat intake on the rectal mucosa [118]. A comparative study revealed that a high-fiber, low-fat diet, similar to that consumed by rural Africans, can have beneficial effects on markers of colon cancer risk, thus decreasing cancer risk in African American patients with chronic inflammation [120]. According to a pilot study, oral beta-glucan derived from Shiitake mushrooms is safe and may increase immune function and quality of life in patients with advanced CRC who are receiving chemotherapy, warranting further investigation in larger, controlled trials [121]. Another RCT revealed that, compared with the control group, the high-fiber group presented a statistically significant reduction in polyp number, whereas the vitamin group presented a nonsignificant trend toward fewer polyps. Researchers have concluded that high-fiber wheat bran supplements can effectively suppress rectal polyp formation in FAP patients, potentially offering a noninvasive strategy for managing this precancerous condition [122].

A Cochrane review assessed the impact of dietary fiber in comparison to a placebo or control on the incidence of colorectal cancer and the recurrence of colorectal adenomatous polyps. The review included 7 RCTs with 4,960 participants, of which 5 studies with 4,798 participants provided data for the meta-analyses and found no statistically significant differences between the control and intervention groups over 2–4 years of follow-up. According to the authors, there is insufficient evidence from current RCTs that consuming more dietary fiber lowers the incidence of CRC or the recurrence of adenomatous polyps [134]. Several significant limitations of the included studies were noted in this review. First, the large number of participants lost to follow-up across the trials introduced significant attrition bias, with the sensitivity analyses showing that the results were not robust to these missing data. Additionally, the RCTs relied on the surrogate outcome of adenomatous polyps rather than the definitive endpoint of colorectal cancer, and the authors caution that the relationship between these two outcomes is not well established. Finally, the RCTs were limited by their relatively brief follow-up periods, which might not have been enough to identify any possible protective effects of dietary fiber against the development of CRC [134].

Dietary fiber was found to be ineffective in reducing the recurrence of colorectal adenoma according to the findings of three RCTs [123,127,128]. The results of another randomized controlled trial showed that a diet high in fiber and low in fat did not lower the risk of colorectal polyp recurrence [124]. Additionally, an RCT revealed that neither wheat bran fiber nor calcium supplementation, alone or in combination, significantly altered fecal bile acid concentrations compared with the placebo; thus, these supplements, at the doses used in the study, did not seem to influence fecal bile acid profiles among patients who had a history of adenomatous polyps [126]. Another randomized controlled trial reported that neither wheat bran fiber nor *L. casei* administration, alone or in combination, effectively prevents the development of new colorectal tumors in this high-risk population [125]. Furthermore, a randomized phase II trial indicated that none of the dietary interventions, whether used alone or in combination, had a significant effect on reducing aberrant rectal crypt foci. The trial also revealed that mucosal proliferation and apoptosis were similar in both the intervention groups and the control group, suggesting that there was no significant effect on cancer-related cellular processes [131].

Here, a question arises as to why there is a lack of convincing evidence from RCTs regarding the prevention and progression of CRC by dietary fiber interventions, even though there is substantial evidence from epidemiological and preclinical studies for the use of the same type of dietary fiber. The reasons could be multiple; one of the important reasons is that the majority of RCTs have been conducted on individual nutrients or specific types of individual fiber, whereas epidemiological studies are generally based on the “dietary pattern” of food consumed (sufficient and regular consumption of fruits and vegetables and whole grains (cereals)) by individuals. Generally, it is widely accepted that the overall pattern of food consumed by an individual is linked to the prevention or onset of certain metabolic diseases or malignancies, including CRC, rather than an individual nutrient. Therefore, there is a need for RCTs that focus on dietary patterns of foods, supported by the findings of observational studies. Such trials will hopefully generate more conclusive results. In addition, there is insufficient understanding of the therapeutically effective dose and the form or type of dietary fiber, as well as the minimum duration of treatment, optimal timing of exposure, and effective endpoint assessment. There is marked variation in these important parameters across different RCTs, which leads to inconsistent findings. Therefore, pharmacokinetic (absorption, distribution, bioavailability, metabolism, elimination) studies that explore the intestinal absorption, transport efficiency and bioavailability of dietary fiber in *in vitro* and *in vivo* animal models are needed.

Another significant concern is that most clinical studies have focused predominantly on individual dietary constituents or one specific kind of dietary fiber, which might not have produced full therapeutic effects. When considering the whole dietary fiber in its natural matrix or when integrating multiple types of fiber, there is potential for synergistic effects that may enhance therapeutic responses. For example, a study demonstrated that whole grains could help prevent the recurrence of adenomas, whereas isolated dietary fiber does not have a comparable effect [135]. This is because whole grains contain several other micronutrients and bioactive compounds, such as polyphenols, folate and a high proportion of vitamin B, and these substances may augment the anticancer properties of dietary fiber. Whole grains and fiber may also provide indirect protection against CRC by reducing the incidence of type 2 diabetes and weight gain, two important risk factors for CRC. This emphasizes how crucial it is to prevent CRC by focusing on whole grains rather than specific ingredients such as fiber supplements [136]. In addition, there are other limitations associated with RCTs, such as poor compliance with the intervention. In dietary interventions, ensuring participant adherence can be particularly challenging. Individuals may struggle to follow dietary recommendations rigorously over extended periods, leading to insufficient data about the true effects of dietary fiber on CRC outcomes. Moreover, the length of follow-up in these studies may be a limiting factor. CRC development is a long-term process, and many RCTs might not have sufficiently prolonged follow-up periods to observe the cancer-related outcomes associated with dietary fiber intake effectively. Additionally, measuring the actual intake and impact of dietary fiber on cancer progression presents challenges. Self-reported dietary assessments can introduce significant bias, making it difficult to ascertain the true relationship between fiber consumption and CRC. The underlying biological mechanisms through which dietary fiber might impact CRC are complex and not entirely understood. This complexity can complicate the interpretation of RCT data. Finally, in dietary intervention trials, achieving true blinding is difficult. The participants were aware of whether they were consuming a high-fiber or low-fiber diet, potentially introducing bias.

RCTs that demonstrate the beneficial effects of dietary fiber provide strong evidence that the interventions utilized to provide protection against CRC. However, if an RCT concluded that there is no effect of dietary intervention, this does not always imply that these interventions are either unrelated or detrimental when considered within the broader context of overall limitations of the conducted studies. Therefore, one should not interpret the findings from intervention human studies as a definitive contradiction to the valuable insights garnered from various types of large-scale and extensive epidemiological studies, supported by compelling data from *in vitro* and *in vivo* animal studies identifying credible biological mechanisms that could explain the observed associations.

## 6. Limitations of the study

The limitations of this narrative review stem from its inherent methodological constraints. Unlike systematic reviews, this narrative approach lacks a comprehensive and reproducible methodology for study selection and analysis, potentially introducing selection bias. The review relies primarily on studies published in English, which may exclude relevant findings in other languages, limiting the breadth of evidence considered. Additionally, while we have summarized key preclinical, epidemiological, and clinical studies, the heterogeneity of methodologies, populations and endpoint assessments in the included research poses challenges for drawing definitive conclusions. The absence of quantitative synthesis, such as meta-analysis, further limits the ability to assess the strength and consistency of the evidence. Finally, the evolving nature of research on dietary fiber and CRC means that some recent developments or unpublished studies may not be captured, underscoring the need for ongoing evaluation of this topic.

## 7. Conclusion

There is convincing evidence from a vast body of epidemiological studies that the intake of dietary fiber prevents CRC. Extensive *in vitro* and animal studies also provide compelling evidence that dietary fiber consumption is not only preventive but also beneficial in treating CRC as adjuvant therapy. Both *in vitro* and animal studies confirmed that dietary fiber has both indirect and direct anticancer effects on CRC through a variety of different molecular and cellular pathways. However, there is limited conclusive evidence from RCTs supporting the effectiveness of dietary fiber in preventing and treating CRC as supportive therapy. The RCTs conducted thus far have several limitations; in particular, there is a lack of standardized doses, formulations, combinations, and durations of exposure to dietary fiber. Large-scale targeted randomized clinical trials that consider the use of combinations of various dietary fiber types with different mechanisms of action to achieve synergistic effects at appropriate therapeutic doses are therefore needed. The findings of such RCTs will guide the health community in the selection of the most effective preventive and adjuvant therapy for CRC. In addition, considering the multiple and diverse benefits of dietary fiber for health and the risk associated with a fiber-deficient diet, it is important to optimize the intake of dietary fiber in our diets, as recommended by multiple international health regulatory authorities, to improve the overall health of the general population.

## Data Availability

Data sharing is not applicable to this article as no new data were generated or analysed in this study.
